# Genetic heterogeneity of actionable genes between primary and metastatic tumor in lung adenocarcinoma

**DOI:** 10.1186/s12885-016-2049-z

**Published:** 2016-01-18

**Authors:** Eun Young Kim, Eun Na Cho, Heae Surng Park, Arum Kim, Ji Young Hong, Seri Lim, Jong Pil Youn, Seung Yong Hwang, Yoon Soo Chang

**Affiliations:** Department of Internal Medicine, Yonsei University College of Medicine, Seoul, 03722 Republic of Korea; Department Pathology, Yonsei University College of Medicine, Seoul, 03722 Republic of Korea; Department of Bio-Nanotechnology, Hanyang University, Sangnok-gu, Ansan, Gyeonggi-do 15588 Republic of Korea; Department of Molecular and Life Science, Hanyang University, Sangnok-gu, Ansan, Gyeonggi-do 15588 Republic of Korea; Bio-Core Co., Guro-gu, Seoul, 08380 Republic of Korea; 8th Floor Annex Building, Gangnam Severance Hospital, Yonsei University College of Medicine, 211 Eonju-ro, 06273 Seoul, Korea

**Keywords:** Genetic heterogeneity, Mutation, Next generation sequencing, Non-small cell lung cancer, Lung adenocarcinoma

## Abstract

**Background:**

Biopsy for lung cancer diagnosis is usually done at a single site. But it is unclear that genetic information at one biopsy site represents that of other lesions and is sufficient for therapeutic decision making.

**Methods:**

Non-synonymous mutations and insertions/deletions of 16 genes containing actionable mutations, and intron 2 deletion polymorphism of *Bcl2-like11* were analyzed in 41 primary tumor and metastatic lymph node (L/N) matched, pStage IIA ~ IIIA non-small cell lung cancer (NSCLC) samples using a next generation sequencing based technique.

**Results:**

A total of 249 mutations, including 213 non-synonymous mutations, 32 deletions, and four insertions were discovered. There was a higher chance of discovering non-synonymous mutations in the primary tumors than in the metastatic L/N (138 (64.8%) *vs.* 75 (35.2%)). In the primary tumors, 106 G > A:C > T transitions (76.8%) of 138 non-synonymous mutations were detected, whereas in the metastatic L/N, 44 (58.7%) of 75 were discovered. A total 24 (11.3%) out of 213 non-synonymous mutations were developed in the context of APOBEC signature. Of those, 21 (87.5%) was detected in the primary tumors and 4 (16.7%) was detected in the metastatic L/N. When the mutation profiles between primary tumor and metastatic L/N were compared, 13 (31.7%) of 41 cases showed discrepant mutation profile. There were no statistically significant differences in disease free survival and overall survival between groups showing identical mutation profiles and those with discrepancy between primary and metastatic L/N.

**Conclusions:**

Genetic heterogeneity between the primary and L/N metastatic lesions is not infrequent finding to consider when interpreting genomic data based on the result of one site inspection. A large prospective study may be needed to evaluate the impact of genetic heterogeneity on the clinical outcomes of NSCLC patients.

**Electronic supplementary material:**

The online version of this article (doi:10.1186/s12885-016-2049-z) contains supplementary material, which is available to authorized users.

## Background

Lung cancer is a leading cause of cancer death worldwide. In 2012, 1,824,701 new lung cancer cases were diagnosed and 1,590,000 patients died of this devastating disease worldwide [1]. The discovery of oncogenic driver mutations has contributed to the determination of the pathogenesis of lung cancer, development of therapeutic target agents, and prediction of treatment responses [2–4]. The identification of actionable oncogenic driver mutations that guide selection of target agents has improved clinical outcomes of lung cancer patients, making tumor genotyping routine clinical practice [5].

However, variable responses and eventual development of resistant clones to target agents are critical challenges to the drug development and therapeutic application. Recent advances from clinical studies have led investigators to posit that targeted therapies may fail to cure on account of tumor heterogeneity. Tumor heterogeneity implies that there are distinct morphological and phenotypic profiles of cellular morphology, gene expression, metabolism, motility, proliferation, and metastatic potential between tumors of the same type in different patients (intertumor), and between cancer cells within a single tumor of a patient (intratumor) [6]. Tumor heterogeneity challenges not only the relevance of driver mutations to the clinical outcome and to response to target therapies, but also the development of diagnostic and therapeutic biomarkers [7–11].

Development of heterogeneous clones inside tumors has been modeled by cancer stem cell (CSC) models and clonal evolution models. In the CSC model, heterogeneity among cancer cells is the result of differences in the stem cells from which they originate, and the variation of stem cells is explained by (1) epigenetic changes and (2) clonal evolution of the CSC population. On the other hand, in the clonal evolution model, tumors arise from a single mutated cell that accumulates diverse mutations with its progression of time in heterogeneous microenvironment. In the clonal evolution model, the spatial differences in exogenous mutational pressure such as cigarette smoking [12–15] and endogenous processes of up-regulation of APOBEC (apolipoprotein B mRNA-editing enzyme, catalytic polypeptide-like) cytidine deaminase activity contribute to the regional variation in mutational burden over time [16–18]. The spatial and temporal diversity in lung cancer evolution is attributed to a decrease in smoking-related mutation accompanied by an increase in APOBEC-associated mutation [15].

The mutagenic effects of cigarette smoking are associated with C > A transversion [14] and the proportion of C > A transversion is decreased in branched (late) mutations, which is indicative of a regional heterogeneous mutational burden attributed to smoking [15]. APOBEC cytidine deaminase is a class of cytidine deaminases that convert cytosine to uracil during RNA editing in the context of a T**C**W motif (where W corresponds to either A or T; the mutated base underlined). APOBEC-associated mutagenesis contributes to tumor progression and a large portion of subclonal diver mutations occur in the APOBEC context, which eventually contributes to a different mutational spectrum across regions and time [19].

We doubt that a biopsy at a single site represents the genetic variation of the entire tumor. To clarify this, genetic profiles of primary tumor and metastatic lymph node (L/N) in the surgically resected pStage IIA-IIIA non-small cell lung cancer (NSCLC) were compared using next-generation sequencing (NGS)-based repeated deep sequencing of 16 genes containing actionable oncogenic mutations. The mutation profile of primary tumors and corresponding metastatic L/N was examined in respect of smoking related and APOBEC mediated signature. The difference in the genetic profiles between lesions was frequently observed in the genes that have important roles in cancer biology and it might influence responses to therapeutic agents.

## Methods

### Patient characteristics and tumor DNA samples

A total of 59 patients who met the following criteria were randomly selected from tissue archives of affiliated hospitals of Yonsei Medical Center: (1) a pathologically confirmed diagnosis of pStage IIA ~ IIIA lung adenocarcinoma, (2) history of curative surgical resection, (3) availability of tissues of both primary tumor and metastatic L/N, (4) confirmation of known oncogenic driver mutation either at *ALK, EGFR*, or *KRAS*, (5) history of platinum-based adjuvant chemotherapy and (6) submission of informed consent for tissue collection. After analysis of NGS data, 18 cases were excluded from further analysis because a driver mutation had not been confirmed at both the primary tumor and metastatic L/N. For the extraction of cancer enriched DNA from tissue block, paraffin-embedded tissue samples were loaded onto silanated slides as 4-μm-thickness sections. One slide of each block was lightly stained with H&E, re-examined for the presence of cancer cells and then the cancer-enriched area was marked by an independent lung pathologist. The cancer cell-enriched areas were scrapped with blades, and DNA was extracted using a QIAamp DNA FFPE Tissue Kit (Qiagen, Valencia, CA, USA). This study was approved by the ethical committee of Gangnam Severance Hospital (IRB #3-2013-0298).

### Library preparation, NGS with Ion Torrent, and variant calling

Ten ng of gDNA was amplified by the Ion AmpliSeq™ Custom Panel (Life Technologies, Carlsbad, CA). This panel contains 16 genes that contain actionable mutations; *AKT1, ALK, BCL2L11, BRAF, DDR2, EGFR, ERBB2, FGFR1, KRAS, MAP2K1, MET, NRAS, PIK3CA, PTEN, ROS1, and RET* [20]*.* The process for generating sequencing data using the Ion Torrent PGM sequencer is described elsewhere**.** Briefly, multiplex pools were purified with Agencourt AMPure XP beads (Beckman Coulter Incorporated) and ligated with Ion Xpress barcode adapters (Life Technologies). The fragment size and quantity of each library were analyzed with a BioAnalyzer using a High Sensitivity Chip (Agilent, Santa Clara, CA). The library was diluted, and emulsion PCR was performed with the OneTouch™ reagent kit (Life Technologies). The emulsion PCR product was enriched using DynabeadsR MyOneTM Streptavidin C1 beads (Life Technologies). The final enriched ion spheres were mixed with a sequencing primer and polymerase and loaded onto five 318v2 chips. The libraries were sequenced with the Ion Torrent PGM sequencer at deep coverage (aiming for 1000×) using the Ion OneTouch 200 Template Kit v2 DL and Ion PGM Sequencing 200 Kit v2 with the 318 v2 chip kits (all from Life Technologies). The sequencing reads were aligned to the human reference GRCh37 genome, and base calling was performed using the Ion Torrent Suite V3.4.2 using tmap-f3 on the Ion Torrent server. The Ion Torrent Variant Caller (ITVC) v3.4 was used for the detection of mutations, requiring a frequency greater than 5% for a variant to be called. BAM (Binary sequence Alignment/Map format) and FASTQ files (alignment) were generated based on the base calling results and were used to report variant calling, including single nucleotide polymorphisms (SNPs) and insertions/deletions (INDELs).

### Statistical analysis

Categorical and continuous variables were compared using χ^2^-tests and *t*-test, respectively. Differences in distribution of continuous variables between two independent samples were assessed by Mann–Whitney U test, and the Kaplan–Meier estimator was used for survival analysis. All analyses were performed with IBM SPSS Statistics version 20 (IBM Corp**.**). All statistical tests were two-sided, and a P**-**value <0.05 was considered to be statistically significant.

## Results

### Demographic characteristics of the study population

**Fig. 1:**
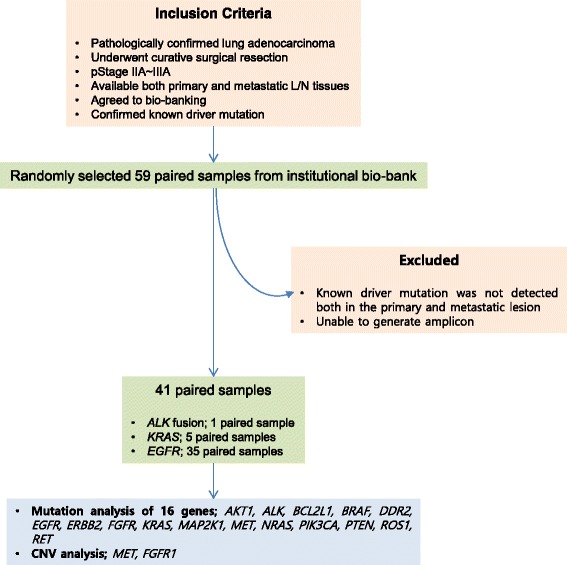
Study diagram. A total of 59 paired primary lung adenocarcinoma and corresponding lymph node metastasis with confirmed driver mutations were randomly selected from the institutional bio-bank. We excluded 18 cases in which the known driver mutation was not detected in both the primary and the metastatic lesion, or in cases where we were unable to generate adequate amplicons for both lesions

The study cases were extracted at random from a subset of the institutional tissue archives for which the cases were confirmed to harbor known *EGFR, KRAS*, and *ALK* driver mutations. This strategy was adopted based on the hypothesis that these driver mutations are trunk mutations that develop early in lung carcinogenesis and propagate throughout cancer progression [11, 15]. The presence of these mutations was used as a marker for the presence of cancer DNA in the test samples and was also used as a reference suggesting that a discovered mutation originated from cancer DNA. If the *EGFR, KRAS*, or *ALK* driver mutation was not confirmed both in the primary and metastatic lesion, the case was excluded from further analysis (Fig. 1). Eighteen of 59 cases that failed to show driver mutations in the metastatic L/N were excluded from analysis based on this prerequisite. Driver mutations in the 41 cases were as follows: *ALK* rearrangement 1, *KRAS* 5, and *EGFR* 35 pairs. The mean age of the study population was 59.7 ± 11.06 years (range, 34–83 years). Thirteen patients (31.7%) were male and 28 (68.3%) were female. There was significant difference in the age at the time of diagnosis between male and female patients. The mean age of females was 57.3 ± 10.72 and that of males was 64.8 ± 10.49 years (P-value = 0.043, *t*-test). The majority of patients (33; 80.5%) did not have a smoking history. Three (7.3%) were current smokers and 5 (12.2%) were ex-smokers (Table 1).

**Table 1:** Clinical and pathologic characteristics of the study cases

			*n* = 41
Age (mean ± SD); yrs			59.7 ± 11.06
Gender			
	Male		13
	Female		28
Smoking status			
	Never smoker		33
	Current smoker		3
	Ex-smoker		5
pStage			
	IIA		17
	IIB		1
	IIIA		23
Driver mutation			
	*ALK* fusion (*n* = 1)		
	*EGFR* mutation (*n* = 35)^a^		
		L858R	16
		L861Q	1
		E19del	19
		E20ins	1
		G719Ser	2
	*KRAS* (*n* = 5)		
		G12V	2
		G12C	1
		G12D	2
Histologic subtypes			
	Lepidic predominant		1
	Acinar predominant		29
	Papillary and micropapillary predominant		8
	Solid predominant with mucin production		2
	Invasive mucinous adenocarcinoma		1

^a^Four cases harbored double *EGFR* driver mutations (See the Additional file 1)

### Primary tumor has a higher mutational burden than metastatic L/N

**Fig. 2:**
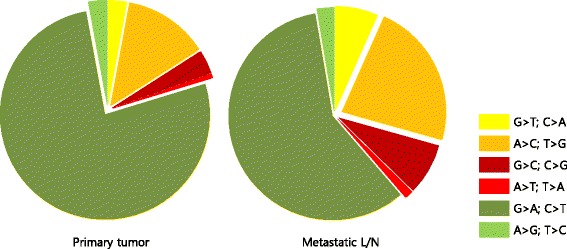
Comparison of relative frequency of non-synonymous point mutations between primary lesion and metastatic lymph nodes

A total of 249 mutations were discovered in the 41 paired primary and metastatic lesions, including 213 non-synonymous point mutations, 32 deletions, and 4 insertion mutations. Another four cases showed germline deletion polymorphism of *Bcl2-like11 (BCL2L11)* intron 2. There were higher chances to discover non-synonymous point mutations in the primary tumor than in the metastatic L/N (138 (64.8%) *vs.* 75 (35.2%)). Both in the primary tumor and metastatic L/N, G > A:C > T transitions contributed to a major portion of non-synonymous point mutations. In the sequencing data of 41 primary tumors, 106 G > A:C > T transitions (76.8%) were discovered out of 138 non-synonymous mutations whereas those of the matched metastatic L/N showed 44 G > A:C > T transitions (58.7%) from 75 non-synonymous mutations, indicating that the proportion of the G > A:C > T transition is higher in the primary tumors than the metastatic L/N. On the other hands, the fraction of transversion mutation was higher in the metastatic L/N than in the primary tumor. In the sequencing data of primary tumor, the transversion mutation that contributed to non-synonymous mutation amounted to 20.3% (28 of 138), whereas in the data from metastatic L/N, it accounted for 40.2% (29 of 75) (Table 2 and Fig. 2).

**Table 2:** Non-synonymous point mutations detected in the primary and metastatic L/N

		Primary tumor	Metastatic L/N	Total
Transversions	G > T: C > A	4	5	9
	A > C: T > G	18	17	35
	G > C: C > G	5	6	11
	A > T: T > A	1	1	2
Transitions	G > A: C > T	106	44	150
	A > G: T > C	4	2	6

APOBEC-mediated mutagenesis is another factor that contributes to the genetic heterogeneity of cancer and more remarkable in the branched mutation than trunk mutation [15]. To compare the contribution of APOBEC-mediated mutagenesis in the primary tumor and metastatic L/N, we evaluated APOBEC mutation signature in the identified non-synonymous mutations. C to T and C to G substitution in T**C**W motif and the complementary W**G**A to WAA or WCA was defined as APOBEC signature mutations [16]. A total 24 (11.3%) out of 213 non-synonymous mutations were developed in the APOBEC signature. Of these, 21 (87.5%) was detected in the primary tumor and 4 (16.7%) was detected in the metastatic L/N, indicating APOBEC mediated mutation signature was more prominent in the primary tumor (Additional file 1).

### Heterogeneity of missense mutation between primary and metastatic lesions is common

To illustrate the spatial heterogeneity of somatic mutation of the 16 genes of the study panel, the individual mutation of the primary tumors and the metastatic L/N are schematized in the Table 3. In this panel depicting genetic variation, non-synonymous mutations that were identical between the primary and metastatic sites are denoted in blue and those that reside either in the primary site or metastatic lesion, indicating discrepancies, are marked in red. Also, different mutations are located in different row of the panel.

**Table 3:**
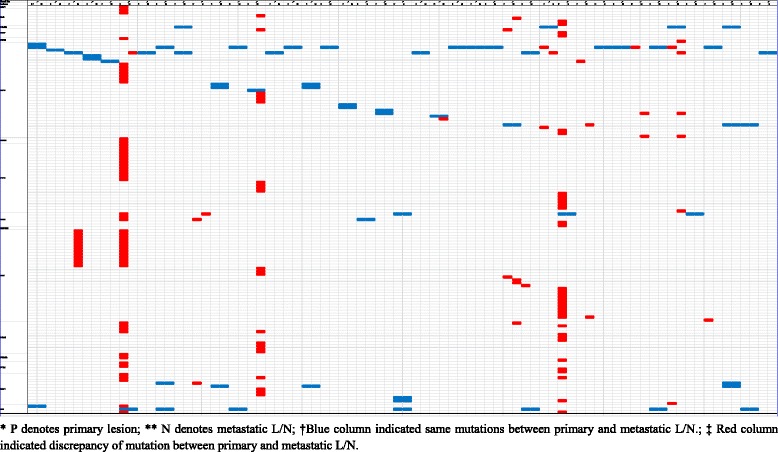
Comparison of mutations between primary and metastatic L/N

From 249 mutations (213 non-synonymous point mutations, 32 deletions, and 4 insertions), 140 different types of phenotypic changes in amino acids were identified. Mutations in *EGFR* accounted for a significant portion of these changes, showing 31 different types of amino acid changes, followed by *MET* 32, *FGFR* 24, *NRAS* and *ROS1* 11 (Table 3 and Additional file 1). As it can be inferred from the characteristics of the study population, *EGFR* had a larger number of mutations than any other genes in this study panel showing a total of 99 somatic mutations in 41 paired samples. Among the detected *EGFR* mutations, 37 mutations were identified both at the primary and metastatic site, whereas 25 mutations were identified either in the primary or metastatic site (Table 3 and Additional file 1). *MET* showed 46 mutations and they were identified either in the primary tumor or metastatic lesions, but not in both lesions, showing a 100% heterogeneous nature of mutation. On the other hand, *MAP2K1* and *BCL2L11* did not have somatic mutations. Twenty-eight (68.3%) of 41 cases showed a mutation profile that was exactly the same between primary and metastatic sites, and 13 cases (31.7%) showed different mutation profiles. In 4 cases, different mutations were discovered either in the primary lesion or metastatic site of paired samples. Another 4 cases showed accumulations of new mutations in the metastatic L/N and 5 cases showed new mutations in the primary tumor. These findings indicate that mutation of the primary lesion is not fully transferred to the metastatic lesion.

**Fig. 3:**
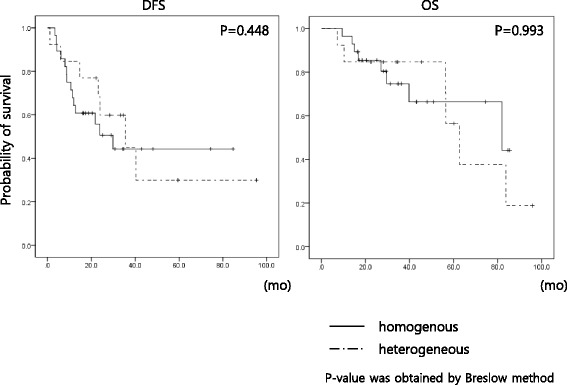
Comparison of disease-free survival (DFS) and overall survival (OS) according to the heterogeneity of non-synonymous point mutations between primary lesion and metastatic lymph nodes. Kaplan–Meier estimation was used to compare the DFS and OS of the study population and there was no difference in the DFS and OS in the study population that underwent curative resection

Demographic and clinical parameters were compared between the group with an identical mutation profile for both primary and metastatic sites and the group with discrepant mutation profiles (Table 4). No tested clinical parameters were significantly different between groups. Next, we analyzed whether the discrepancy in mutations between the primary tumor and metastatic site influenced the clinical outcome of the study population. When the disease free survival (DFS) and overall survival (OS) was compared between groups showing identical mutation profiles and those with discrepancy between primary and metastatic lesions, there were no statistically significant differences in DFS and OS (Fig. 3). Collectively, differences in the mutation profiles between primary tumor and metastatic site were common and the mutation of the primary site was not always transferred to the metastatic lesion. Also, these clinical parameters could not be used to gauge spatial heterogeneity of lung cancer.

**Table 4:** Clinical and pathologic characteristics of the study cases according to the heterogeneity of mutation

		Homogenous (*n* = 28)	Heterogeneous (*n* = 13)	*P*-value
Age (mean ± SD); yrs		59.7 ± 12.31	59.6 ± 8.17	0.996*
Gender	Male	9	4	0.930**
	Female	19	9	
Smoking status	Never smoker	22	11	0.132**
	Current smoker	1	2	
	Ex-smoker	5	0	
pStage	IIA	12	5	0.331**
	IIB	0	1	
	IIIA	16	7	
Maximum tumor diameter (cm)		3.0 ± 1.02	3.28 ± 1.15	0.401*
Histologic subtype	Lepidic predominant	0	1	
	Acinar predominant	20	9	0.732**
	Papillary and micropapillary predominant	6	2	
	Others^a^	2	1	

*P-value was obtained from t-test; **P-value was obtained from Pearson’s Chi-square test; ^a^includes solid types and invasive mucinous adenocarcinoma

## Discussion

Despite the continuous development of new therapeutic modalities for lung cancer, the outcomes still fall short of our expectations and a cure is seldom observed in patients treated with targeted therapy. One possible explanation for the heterogeneous responses and eventual development of resistance clones to the target agents is the existence of various types of tumor heterogeneity. Initial phenotypic heterogeneity and changes in cellular phenotypes resulting from adaptation to response and selection for resistant phenotypes need to be accounted for in order to achieve substantial improvements in therapeutic outcomes [11]. At the same time, given the link between genetic heterogeneity and poor prognosis, a measure of heterogeneity by itself may be useful as a prognostic marker [11].

In this study, spatial genetic heterogeneity of lung adenocarcinoma was compared by analyzing differences in 16 genes with known actionable mutations between primary and metastatic L/N. NSCLC tissues with confirmed *EGFR, KRAS,* and *ALK* driver mutations were randomly selected from our institutional tissue archives. The studies from the East Asian NSCLC patients showed that the mutation profile of East Asian NSCLC is different from that of Western population. Excluding the cases without identifiable driver mutations, the mutation profile of this study cases was *EGFR* 85.3%, *KRAS* 12.2%, and *ALK* break-apart 2.4 %, which is comparable to the previous reports from East Asia. One study that recruited 1420 NSCLC patients revealed that 82 (5.8%) cases harbored *KRAS* mutations either in codon 12 or 13. The low frequency of *KRAS* mutation and high *EGFR* mutation rate is consistent to the other reports from East Asia [21–25]. When a confirmed driver mutation was not validated in both the primary lesion and metastatic lesion by the study panel, it was excluded from further analysis, based on the concept that these known driver mutations occur early in cancer development and further propagates through branched clonal evolution [15, 26–28]. Using this criterion, 18 of 59 (30.5%) pairs were excluded from analysis. This simple but conservative study design may harbor risks that it might miss new clinically relevant mutations in the subclones. However, it might be a useful indicator of the presence of cancer DNA in the tissue samples mixed with a small number of cancer cells. Indeed, a recent meta-analysis on the differences in *EGFR* and *KRAS* mutation between primary and corresponding metastatic NSCLC tumors indicated that there is no difference between lesions [26]. Compared to genome-wide approaches, this study adapted a focused approach with a specific set of genes that contain actionable mutations [6]. We also adapted sequencing of pooled DNA from regional sampling instead of single cell sequencing, or longitudinal sampling approaches [11, 29–31]. This approach has limitations to the analysis and classification of heterogeneity that may be attributed to diverse causes, but it may be the most relevant method applicable to the current medical fields.

In the primary tumor of the current smoker, where the mutagenic pressure of cigarette smoking is stronger than that of metastatic site, the proportion of C > A transversion is higher than that of the metastatic sites [14, 15]. Interestingly, this study revealed that C > A transversion was more frequently detected in L/N metastasis rather than primary tumor, suggesting that other mechanisms that cause heterogeneity exist even considering the majority of study population are composed of non-smokers. The APOBEC deaminase activity which result in increase of C > T:G > A and C > G:G > C mutation in the context of T**C**W motif may be attributed to the different regional accumulation of missense mutation with cancer progression [15]. This study applied less stringent condition of APOBEC mediated mutation and showed that APOBEC signature is more prominent in the primary tumor rather than metastatic L/N. This can be assumed that the primary tumor has larger tumor burden with more complex microenvironment than the metastatic site.

Genetic heterogeneity may be implicated in poor clinical outcome. This study population did not show differences in the DFS and OS between those with homogenous mutation profile and those with heterogeneous mutation profile. It may originate from that the study population composed of pStage IIA-IIIA NSCLC patients treated with curative surgical resection and adjuvant chemotherapy based on platinum. Further studies are required on the patients with stage IV NSCLC who are treated with target agents.

Spatial heterogeneity, indicating that a biopsy of single site does not represent the whole biology of cancer, may pose significant challenges in the personalized medicine. The cases with large tumor burden and those with treatment failure require profound consideration for the number and location of target biopsy lesion, which guide treatment planning and improve the clinical outcome of the patients. It needs to be determined which approaches to genetic heterogeneity lead substantial improvement in clinical outcome in an economical way.

## Conclusions

There was pronounced genetic heterogeneity in mutations between the primary and corresponding lymph node metastasis. The regionally separated mutations may compromise treatment success with a target agent in NSCLC. It would be feasible to develop therapeutic strategies based on a common denominator for the whole tumor, rather than targeting a specific mutation in a gene.

## Additional file

Additional file 1: Table S1. Comparion of individual mutations between primary lesion and metastatic L/N. (XLSX 20 kb)

## Abbreviations

APOBEC: Apolipoprotein B mRNA-editing enzyme, catalytic polypeptide-like
CSC: Cancer stem cell
DFS: Disease free survival
INDELs: Insertions/deletions
NGS: Next-generation sequencing
NSCLC: Non-small cell lung cancer
OS: Overall survival
SNPs: Single nucleotide polymorphisms
